# A Case of Axial Spondyloarthritis Triggered by SARS-CoV-2 Infection

**DOI:** 10.7759/cureus.22860

**Published:** 2022-03-05

**Authors:** Joshua Rui Yen Wong, Luke Zhu, Sachi Shah, Srinivas Gadikoppula

**Affiliations:** 1 Trauma and Orthopaedic Surgery, North Middlesex University Hospital, London, GBR

**Keywords:** spondyloarthritis, ankylosing spondylitis, sars-cov-2 (severe acute respiratory syndrome coronavirus-2), axial spondyloarthritis, covid-19

## Abstract

Ever since the outbreak of severe acute respiratory syndrome coronavirus 2 (SARS-CoV-2), there has been a noticeable change in atypical presentations of several rheumatological diseases following COVID-19 infections. In this case report, we present a case of SARS-CoV-2-induced axial and peripheral spondyloarthritis. This case highlights the possibility of SARS-CoV-2 infection accelerating the onset of autoimmune diseases such as axial spondyloarthritis. Although uncommon, these cases warrant a referral to the rheumatologist for appropriate diagnosis and management. This case also highlights the need for further research on the mechanisms behind the viral interaction of SARS-CoV-2 infections with the host immune system, especially about accelerating the onset of autoimmune diseases.

## Introduction

Spondyloarthritis is an umbrella term that includes a spectrum of chronic inflammatory diseases including ankylosing spondylitis, reactive, psoriatic and enteropathic arthritis [1]. The aetiology of spondyloarthritis remains mostly idiopathic, although there is an association with the human leukocyte antigen B27 [2]. While spondyloarthritis usually involves the spine and sacroiliac joints, peripheral joints can also be affected [3]. The outbreak of severe acute respiratory syndrome coronavirus 2 (SARS-CoV-2) triggered a global pandemic of atypical respiratory infections (COVID-19). While SARS-CoV-2 is not known to directly involve skeletal muscle, joints or bones, arthralgia and myalgia are recognised symptoms in COVID-19 cases [4]. In a Spanish case series of 306 proven COVID-19 patients, arthralgias and myalgias were reported in approximately 25% of the clinical presentation [5]. Although arthralgia and myalgia are also common symptoms in other acute viral illnesses, the development of arthritis in COVID-19 specifically has not yet been confirmed to date. However, there has been a noticeable change in atypical presentations of several other rheumatological diseases such as myositis and psoriatic arthritis following COVID-19 infections [6]. Recent reports have also suggested an association between COVID-19 and autoimmune diseases [7]. In this case report, we present a case of SARS-CoV-2-induced axial and peripheral spondyloarthritis.

## Case presentation

A 66-year-old Caucasian male patient presented with a six-week history of worsening lower back pain and a four-week history of atraumatic right-side knee pain and swelling on a background of resolved COVID-19 infection, hypertension and gout well controlled with allopurinol. His previous records showed a positive result for SARS-CoV-2 RNA on real-time polymerase chain reaction using nasopharyngeal swabs approximately a month before the development of his back pain.

On the day of admission, his back pain was compounded by a six-day history of acute faecal constipation and three days of difficulty passing urine. In addition, the patient had reported an unintentional weight loss of approximately 12.7 kg over the preceding two weeks. On further enquiry, the patient declined to have any other gastrointestinal symptoms or pre-existing conditions such as ankylosing spondylitis, benign prostate hyperplasia, psoriasis, uveitis, inflammatory bowel disease, dysuria, mouth ulcers or genital or gastrointestinal infections. He also denied any use of analgesic, recent changes in regular medication, recent acute injury, foreign travel or insect bites.

Clinical observations at presentation were: a respiratory rate of 18 breaths per minute, oxygen saturation of 97% on room air, heart rate of 93 bpm, blood pressure of 157 mmHg systolic and 85 mmHg diastolic and a temperature of 37.6^o^C.

Examination of the patient’s right knee revealed a hot, swollen non-erythematous joint that was maximally tender in the popliteal fossa while being able to bear weight. Examination of the spine revealed tenderness over both sacroiliac joints, reduced lumbar forward flexion and a positive Schober’s test.

**Figure 1:**
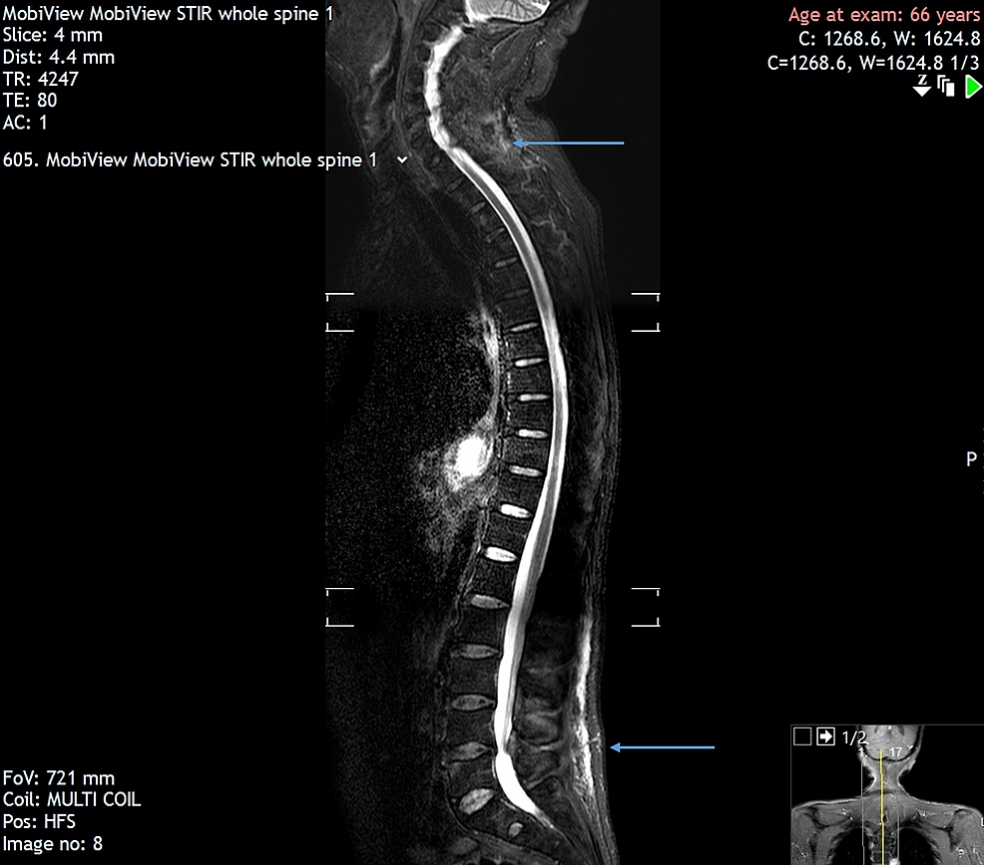
MRI showing facet joint spondylitis at L4-5 and inflammatory changes around the C6 spinous process

Given the patient’s initial presentation of lower back pain in conjunction with urinary and faecal changes, a detailed neurological examination and an MRI scan were performed, which excluded cauda equina syndrome. Nonetheless, the MRI report highlighted facet joint spondylitis at L4-5 with excessive oedema as well as inflammatory changes around the C6 spinous process as seen in Figure 1. No previous MRI was available to make a comparison.

Blood results during the admission were largely normal, except for an elevated serum C-reactive protein (CRP) level of 244 mg/L, an elevated white cell count of 10.42x10^9^/L and an elevated platelet count of 533x10^9^/L, as seen in Table 1.

**Table 1:** Laboratory findings during the admission WBC, white blood cells; CRP, C-reactive protein; eGFR, estimated glomerular filtration rate.

	Day 2	Day 3	Day 8	Reference ranges
Haemoglobin (g/L)	124	118	122	130–170 (male)
WBC (x10^9^/L)	10.42	9.87	17.25	3.0–10.0×10^9^
Platelet count (x10^9^/L)	533	555	609	150–400×10^9^
CRP (mg/L)	244.5	222.2	179.0	<5
Sodium (mmol/L)	141	141	138	135–146
Potassium (mmol/L)	4.5	4.6	4.9	3.5–5.3
Urea (mmol/L)	6.1	5.9	5.4	2.5–7.8
Creatinine (mmol/L)	62	56	53	60–120
eGFR (mL/min/1.73m^2^)	>90	>90	>90	>60

The right knee joint aspirate was found to be sterile (negative knee aspirate microscopy, culture and 16S PCR screen) with scanty white cells present albeit with no evidence of crystal deposition.

Following an inpatient rheumatology review, it was observed that the profoundly elevated C-reactive protein (CRP) was excessive for an inflammatory process and therefore a septic screen was performed to rule out any infections. This included blood cultures, CT scan of the chest, abdomen and pelvis, transthoracic echocardiogram and urine analysis (which all returned negative).

With regard to management, oral nonsteroidal anti-inflammatory drugs were prescribed for analgesia in the first place. The patient was also started on Movicol "full-strength" sachets that quickly resolved acute constipation. Once an infective pathology was excluded, the patient was started on a 30-mg tapering dose of prednisolone on day eight of admission. The patient responded well after review from the pain team reporting marked improvements in his pain and stiffness and hence was subsequently discharged from the hospital on day nine of his admission.

## Discussion

This case highlights the possibility of SARS-CoV-2 infection accelerating the onset of an autoimmune disease such as axial spondyloarthritis. Although current literature reports multiple cases of acute reactive arthritis developed after the resolution of COVID-19 symptoms [8-14], these patients did not report any axial involvement. To date, there is only one reported case of axial spondyloarthritis triggered by SARS-CoV-2 infection [15]. The pathophysiology behind virus-induced arthritis is believed to be a result of multiple mechanisms triggered by the infiltration and formation of immune complexes specifically in the joint spaces, ruling out arthralgia and arthritis [10]. Recently, enriched plasmacytoid dendritic cells have been found at the normal human enthesis [16]. These entheseal plasmacytoid dendritic cells, through stimulation of the toll-like receptor (TLR)-7 and TLR-9, secrete inflammatory cytokines such as tumour necrosis factor (TNF) and type-I interferons via the nuclear factor kappa B (NFκB) or myeloid differentiation factor 88 (MYD88) pathways [17,18]. Given that these plasmacytoid dendritic cells are crucial cytokines in enthesis, this may explain the mechanistic link between SARS-CoV-2 infection and axial spondyloarthritis [16]. Given the novelty of the disease, there is a lack of definitive evidence behind the exact pathophysiology of this phenomenon. Hence, further research must be conducted on the specific mechanisms underlying the viral interaction with the host immune system, especially regarding the potential association between SARS-CoV-2 infection accelerating the onset of autoimmune diseases. Although uncommon, such cases warrant the special attention and expertise of a rheumatologist for appropriate diagnosis and management. Once vital differentials such as cauda equina syndrome, infection and crystal arthropathies are ruled out, COVID-19-induced axial spondyloarthritis should then be considered as a diagnosis of exclusion.

## Conclusions

In this case report, we present a case of SARS-CoV-2-induced axial and peripheral spondyloarthritis. This case highlights the possibility of SARS-CoV-2 infection accelerating the onset of autoimmune diseases such as axial spondyloarthritis. Although uncommon, these cases warrant a referral to the rheumatologist for appropriate diagnosis and management. This case also highlights the need for further research on the mechanisms behind the viral interaction of SARS-CoV-2 infections with the host immune system, especially about accelerating the onset of autoimmune diseases.
